# Inhibitory and preventive effects of *Lactobacillus plantarum* FB-T9 on dental caries in rats

**DOI:** 10.1080/20002297.2019.1703883

**Published:** 2019-12-25

**Authors:** Qiuxiang Zhang, Sujia Qin, Yin Huang, Xianyin Xu, Jianxin Zhao, Hao Zhang, Wei Chen

**Affiliations:** a State Key Laboratory of Food Science and Technology, Jiangnan University, Wuxi, Jiangsu, P. R China; b School of Food Science and Technology, Jiangnan University, Wuxi, Jiangsu, P. R. China; c International Joint Research Laboratory for Probiotics, Jiangnan University, Wuxi, Jiangsu, P.R. China; d Department of Stomatology, Wuxi Children’s Hospital, Wuxi, Jiangsu, P.R. China; e National Engineering Research Center for Functional Food, Jiangnan University, Wuxi, Jiangsu, P.R. China; f Beijing Innovation Centre of Food Nutrition and Human Health, Beijing Technology and Business University (BTBU), Beijing, P.R. China; g Wuxi Translational Medicine Research Center and Jiangsu Translational Medicine Research Institute Wuxi Branch, Wuxi, P.R. China

**Keywords:** Caries, *Streptococcus mutans*, dental plaque, biofilm, probiotics

## Abstract

*Streptococcus mutans* is recognized as the main cause of dental caries, and the formation of a plaque biofilm is required for caries development. This study aimed to determine the inhibitory effect of *Lactobacillus plantarum* FB-T9 on *S. mutans* biofilm formation *in vitro* and on the prevention and treatment of dental caries in rats. During *in vitro* experiments, FB-T9 exhibited good bacteriostatic ability in a plate competition assay. This strain also significantly reduced the biomass and viability of *S. mutans* biofilms and induced structural damage during the early (6 h), middle (12 h) and late (24 h) stages of biofilm formation. In a 70-day *in vivo* experiment, FB-T9 significantly reduced the levels of *S. mutans* on the dental surfaces of rats by more than 2 orders of magnitude of the levels in the dental caries model group (p < 0.05). Moreover, FB-T9 significantly reduced the caries scores (modified Keyes scoring method) in both the prevention and treatment groups (p < 0.05) and had great colonization potential in the oral cavity. These results indicate the potential usefulness of *L. plantarum* FB-T9 as a probiotic for the prevention and treatment of caries.

## Introduction

Caries is a common type of oral bacterial infectious disease. According to the World Health Organization (WHO), caries is the third most common non-communicable disease after cancer and cardiovascular disease. According to data from a previous *Lancet* publication [1], dental caries of the permanent teeth was the most prevalent disease afflicting humans. In 2016, caries in permanent teeth and caries in deciduous teeth ranked second and fifth, respectively, among the 10 diseases with the highest global incidence.

The formation of a plaque biofilm of bacteria is a prerequisite for the occurrence of a caries. A mature dental plaque biofilm is a three-dimensional micro-ecological environment comprising various bacteria embedded in a matrix mainly composed of water-insoluble polysaccharides with a certain thickness. In a normal dental plaque biofilm, oral microflora exist in a dynamic equilibrium. When host, dietary or microbial growth factors alter the ecological balance, acidifying bacteria in the plaque decrease the pH of the biofilm environment. Subsequently, the demineralization–remineralization balance in the tooth shifts toward mineral loss, leading to tooth decay [2]. *Streptococcus mutans*, which has strong acid-producing and acid-resistant capacities, has been recognized as the main type of cariogenic bacteria [3]. A large number of extracellular polysaccharides synthesized by *S. mutans* are important to the complex tri-dimensional structure of a dental plaque. Indeed, many studies have reported the relationship between the presence of *S. mutans*, biofilm formation and the related risk of caries [4–6].

Currently, dental plaque is mainly controlled and prevented using mechanical removal techniques and antimicrobial agents. However, tooth brushing, the most common mechanical plaque removal method, cannot fundamentally reduce the number of cariogenic bacteria such as *S. mutans*. Indeed, the applications of some mechanical treatments in pediatric and elderly populations are limited by personal preferences, and the effects are relatively superficial. The use of antibiotics is also limited by the close structure of dental plaque biofilm that makes the bacteria embedded in this complex community more resistant to antibiotics compared with the planktonic state. Accordingly, the concentration of antibiotics required to kill bacteria in a biofilm is hundreds of times higher than that required to kill planktonic bacteria [7]. In addition, the long-term use of antibiotics may destroy the balance of the oral ecosystem and increase bacterial resistance. Therefore, research into caries prevention and treatment fundamentally relies on a safe and effective caries prevention method.

Probiotics have long been thought to contribute to human gastrointestinal health. To date, studies have confirmed that probiotics can help to prevent and treat diarrhea due to rotavirus infection, antibiotic use, ulcerative colitis and pouch enteritis [8,9]. Probiotics can also prevent respiratory tract infections and osteoporosis in middle-aged women, regulate the balance of oral flora and prevent dental caries, periodontitis and other oral diseases [10–12].

Previous studies have demonstrated that *Lactobacillus* could inhibit the growth of *S. mutans* biofilm and caries-related multispecies biofilms *in vitro* [13–15]. Clinical data have revealed the ability of some probiotics to colonize the oral cavity [16] and significantly reduce the oral population of *S. mutans*. Lee and Kim found that *Lactobacillus rhamnosus* LGG suppressed *S. mutans* biofilm formation by reducing glucan production and antimicrobial activity [17]. Skim soy milk fermented with *L. paracasei subsp. paracasei* NTU101 could considerably reduce the population of *S. mutans* in rats and inhibit the development and deterioration of dental caries [18]. Hence, the screening of candidate oral probiotics based on their abilities to inhibit *S. mutans* growth and biofilm formation may represent an alternative means of preventing tooth decay.

In our previous study, *L. plantarum* FB-T9 isolated from healthy human feces exhibited an excellent antibacterial ability against *S. mutans*. In this study, we evaluated the antibacterial ability of *L. plantarum* FB-T9 on a half-strength plate and investigated the effect of this strain on *S. mutans* biofilm formation over different time periods. We also examined oral *S. mutans* colonization and caries formation in rats using bacterial counts and caries scores to evaluate the effect of *L. plantarum* FB-T9 on the prevention or treatment of caries *in vivo*. These results will support and inform the development of probiotics to prevent caries.

## Materials and methods

### Bacterial strains

*L. plantarum* FB-T9 was isolated from healthy human feces and inoculated in De Man, Rogosa and Sharpe (MRS) broth (Difco^TM^, Detroit, MI, USA) under anaerobic conditions at 37°C. *S. mutans* ATCC 25,175 was purchased from the China Common Microbial Species Preservation and Management Center (CGMCC, Beijing China) and cultured in Tryptic Soy Broth (TSB, Difco^TM^, Detroit, MI, USA) under anaerobic conditions at 37°C. Another strain, *L. plantarum* 5D-3 (5D-3), was selected from a healthy oral cavity and used as a control strain in animal tests. All strains were frozen in 30% (v/v) glycerol broth at −80°C and routinely streaked on corresponding ager plates. The plates were cultured in an anaerobic incubator (Electrotek AW500SG, England) at 37°C for 48 h. A single colony was inoculated into 5 mL corresponding broth tubes anaerobically at 37°C for 16 h and sub-cultured twice using 2% (v/v) inoculum in 5 mL broth before use.

### Competition assay on plate

The experimental method broadly followed Tong et al. [19]. After 16 h culture in broth tubes, the bacterial cultures were centrifuged at 3000 × g for 10 min and washed twice with sterile saline solution. The bacteria were then re-suspended in saline solution and diluted to a suspension with 1 × 10^5^ living cells by colony counting prior to use. FB-T9 and *S. mutans* (10 µL of each) were inoculated on tryptic soy ager plates (TSA) or half-strength TSA (1/2TSA) simultaneously or sequentially. Under the former condition, 10 µL of both strains suspension was inoculated simultaneously and co-cultured at 37°C for 32 h. Under the latter condition, 10 µL of the first colonizer (FB-T9/*S. mutans*) was inoculated and grown for 16 h, after which 10 µL of the second colonizer (*S. mutans*/FB-T9) was inoculated near to the first organism and incubated for another 16 h. All the plates were cultured anaerobically at 37°C.

### Biofilm formation assay

A biofilm formation assay was conducted as proposed by Loo, with slight modification [20]. After 16 h culture, FB-T9 and *S. mutans* were centrifuged and re-suspended in corresponding medium supplemented with 0.2% sucrose, then diluted to a concentration of 1 × 10^5^ CFU/mL by colony counting as described above. Next, a 100-μL aliquot of *S. mutans* suspension was added to each well of a 96-well microplate (Costar, USA) and incubated at 37°C for 24 h. To test the intervening effects of FB-T9 on the formation of the *S. mutans* early-stage biofilm, 40 μL of FB-T9 suspension were added to each well at 0, 6 or 12 h and cultured to a total time interval of 24 h. To assess the effects of FB-T9 on *S. mutans* middle- and mature-stage biofilms, *S. mutans* was initially cultured for 24 or 48 h. After removing the supernatant, 200 μL of FB-T9 suspension was added, and the cultures were incubated for another 24 h. Subsequently, planktonic bacteria were gently aspired from the microplates, which were rinsed with sterile physiological saline solution. Each well was then stained with 200 μL of crystal violet for 30 min at room temperature. After two rinses with saline solution, 100 μL of 95% alcohol was added to release the dye. Biofilm formation was then quantified by measuring optical density at 600 nm (OD_600_) in each well using a microplate reader (BioTek, Winooski, VT, USA). The negative control group was treated with the same volume of saline instead of FB-T9 suspension.

### Biofilm viability assay

An *in vitro* biofilm model was established using a cover slide as carrier [21]. A sterile cover glass was placed in a glass Petri dish with a 6-cm diameter, to which 4 mL of a *S. mutans* suspension was added. At 0, 6 or 12 h, 200 μL of FB-T9 suspension was added to the dish, and the culture was incubated up to a total of 24 h. Otherwise, a *S. mutans* biofilm was allowed to form for 24 or 48 h, after which 4 mL of FB-T9 suspension was added. The culture was then incubated for another 24 h before the biofilm was washed twice with phosphate-buffered saline. Finally, the glass slides were removed and subjected to fluorescence staining as described by Khan [22]. Carboxyfluorescein diacetate, succinimidyl ester (CFDA-SE, 65-0850-84, Invitrogen^TM^) was used to label all viable bacteria with green fluorescence, while propidium iodide (PI, P3566, Invitrogen^TM^) was used to label in red all bacteria with damaged membranes (i.e., non-viable bacteria). The biofilm structure was observed using a confocal laser scanning microscope (CLSM; LSM 710, Zeiss, Germany), and the Z-stack analysis was performed using Zen 2010 software (Carl Zeiss, Germany) [23]. The areas of viable and non-viable bacteria and the biofilm thickness were recorded. The biofilm activity was calculated as the percentage of viable bacteria. All control groups were administered an equivalent volume of saline instead of FB-T9 suspension.

### Caries reduction *in vivo*

#### Animals and general procedures

**Figure 1.**
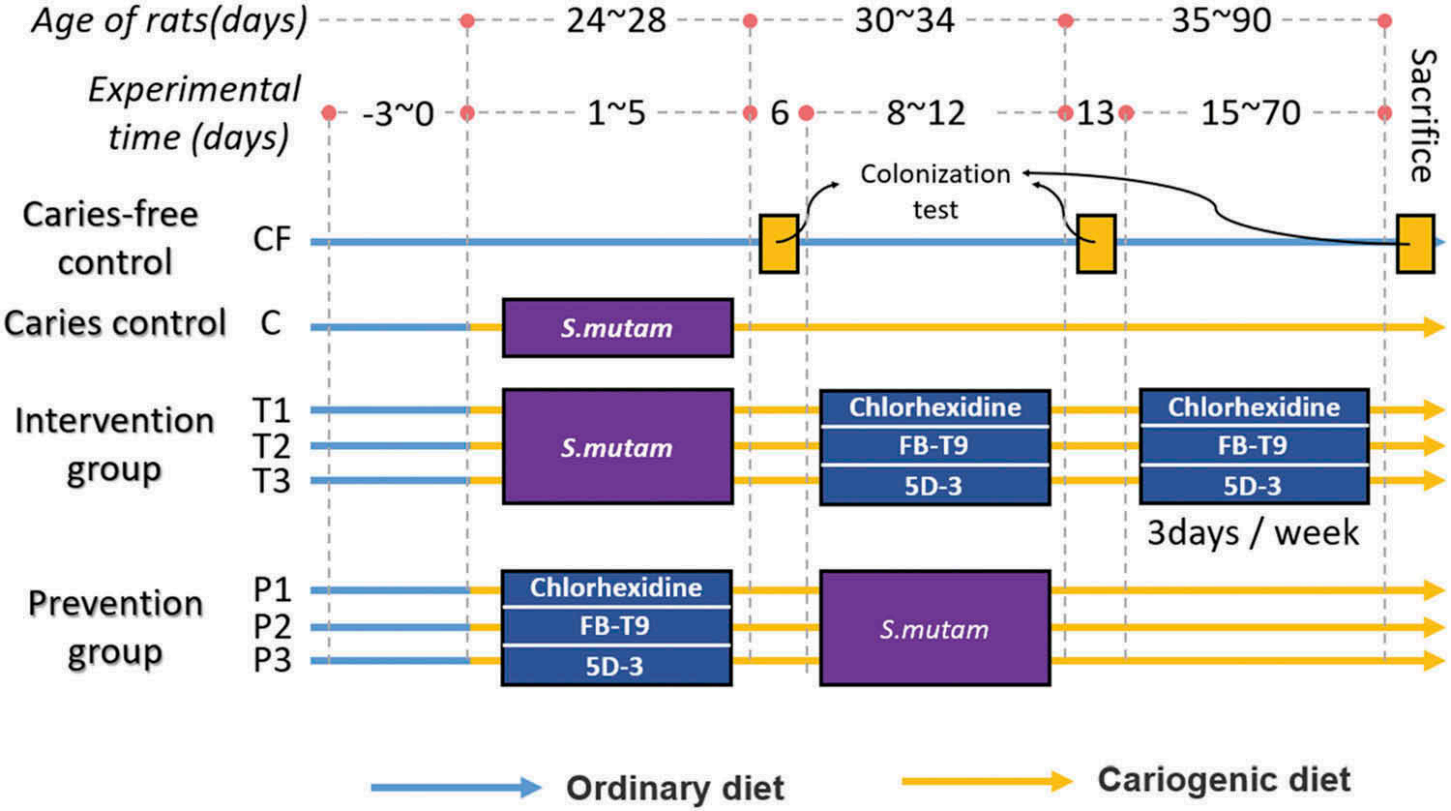
Experimental design for evaluating the induction of dental caries in SPF rats

Female SPF Wistar rats (21 days old) were purchased from Charles River Laboratories (Beijing, China) and reared with approval according to the guidelines of the Animal Care and Use Committee at the Jiangsu Institute of Parasitic Diseases. And the procedures were carried out in accordance with European Community guidelines (Directive 2010/63/EU) for the care and use of experimental animals. All of the rats were allowed to adapt to the diet and environment for 3 days and were then divided randomly into eight groups, comprising three treatment groups, three prevention groups and one group each for the naïve controls and caries models. A flow chart of group allocation is shown in Figure 1. The rats in the dental caries control group (C) were subjected to 5 consecutive days of dental caries modeling. Briefly, after 16 h culture, *S. mutans* cultures were centrifuged and diluted to 10^8^ CFU/mL in saline solution. A sterile cotton swab saturated with this bacterial suspension then applied to the rat oral cavity for 15 s per quadrant, as described by Beiraghi et al. [24]. On day 6, oral swabs were spread on mitis salivarius agar plates supplemented with 200 μg/mL streptomycin sulfate (MMS, Difco^TM^, Detroit, MI, USA) to confirm S. *mutans* colonization in the oral cavity. The intervention groups were treated with chlorhexidine (0.02%) (T1), FB-T9 (T2) or 5D-3 (T3) for another 5 consecutive days after *S. mutans* colonization and thrice weekly thereafter. The prevention groups (P1 0.02% chlorhexidine treated; P2 FB-T9 treated; and P3 5D-3 treated) were first colonized with 10^8^ CFU/mL of lactobacilli for 5 consecutive days and then infected with *S. mutans* for another 5 consecutive days. The caries-free control group (CF) consumed a regular diet and water throughout the 10-week experimental period. All of the other groups were fed a cariogenic diet 2000 (obtained from Nantong Trophy Feed Technology Co., Ltd.) supplemented with a 5% (w/v) sucrose solution.

#### Microbial analysis and caries scoring

The rats were weighed daily to monitor growth in each group. During the experiment, the rats were sampled on days 6, 13 and 70. The collected samples were plated on two types of agar [Tanzer et al., 2010]: MMS agar plate was used to enumerate *S. mutans* against streptomycin, while MRS supplemented with 12 μg/mL vancomycin was used to enumerate lactobacilli [Montella et al., 2013]. At the end of the 10-week experimental period, the rats were anesthetized and sacrificed. The heart, liver, spleen, lungs and kidneys were harvested and weighed. After decapitation, the skull was removed and placed in an autoclave at 121°C for 15 min. The attached soft tissue was peeled off with a scalpel, and the jaw was cleaned and dried at room temperature. All of the specimens were immersed in a 0.4% ammonium purpurate staining solution for 12 h, rinsed and semi-sectioned along the occlusal surfaces of maxillary and mandibular molars using a diamond cutter (thickness: 0.1 mm). Caries on the rat molars was observed and evaluated under a stereomicroscope according to the caries diagnosis and scoring method reported by Keyes [25].

### Statistical analysis

SPSS Statistics 22.0 (SPSS, Inc., Chicago, IL, USA) was used for the analysis. A single factor variance was used to analyze the experimental results (one-way analysis of variance). A p-value <0.05 was considered to indicate a statistically significant difference. Origin Pro 8.5 was used to map and analyze the data.

## Results

### Antagonism between *L. plantarum* and *S. mutans* on a growth plateFB-T9

**Figure 2.**
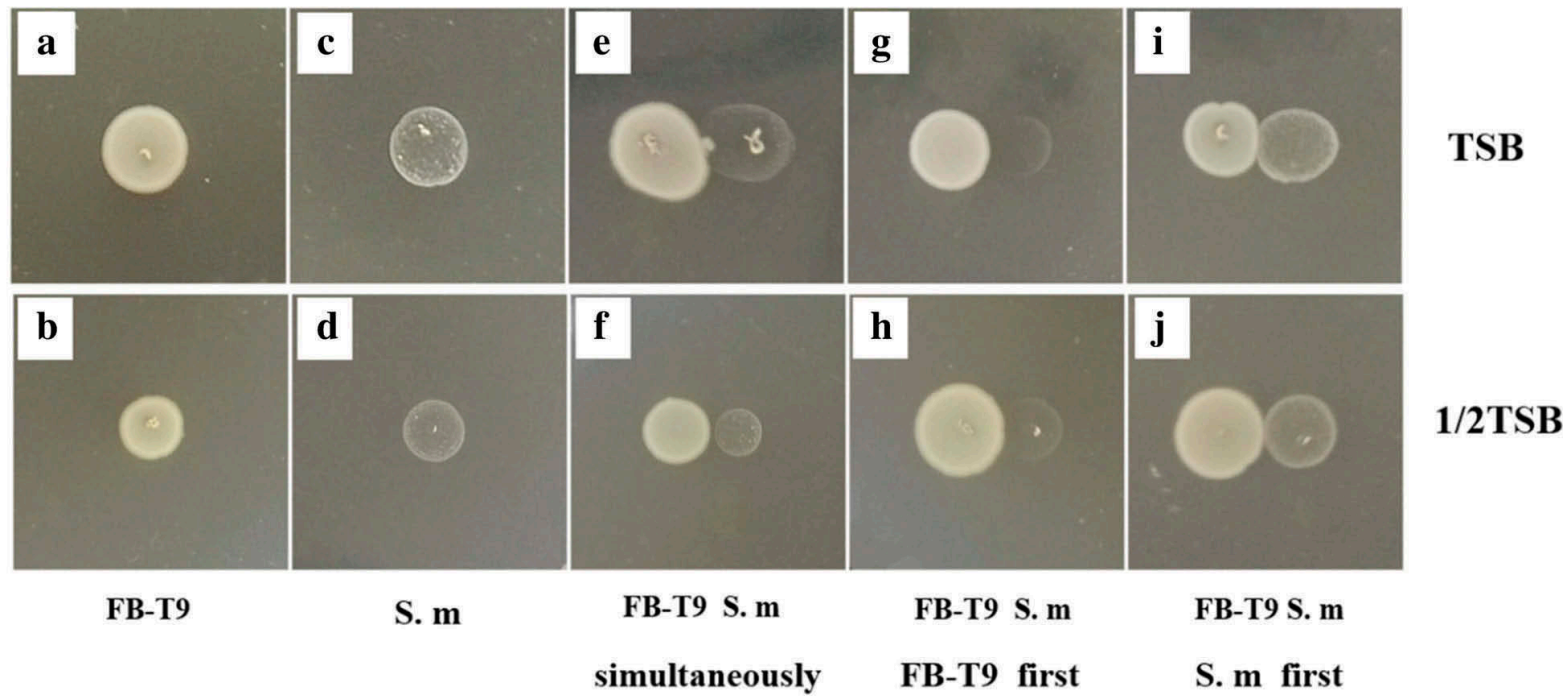
The inhibition effect of *L. plantarum* FB-T9 on *S. mutans*. (A and B) *L. plantarum* FB-T9 on TSA and 1/2 TSA plates; (C and D) *S. mutans* on TSA and 1/2TSA plates. Competition between FB-T9 and *S. mutans*: (E, G, I) on TSA plates; (F, H, J) on 1/2 TSA plates; (E and F) *S. mutans* and *L. plantarum* FB-T9 were inoculated on the plates at the same time; (G and H) *L. plantarum* FB-T9 was first inoculated on the plates; (I and J) *S. mutans* was first inoculated on the plates

A plate competition experiment was used to evaluate the competitive effects of FB-T9 and *S. mutans* in an *in vitro* environment with limited space and nutrients. Both FB-T9 and *S. mutans* grew well on TSA and 1/2TSA plates (Figure 2(a–d)). When FB-T9 and *S. mutans* were spotted on TSA plates simultaneously, the growth of *S. mutans* was suppressed near the FB-T9 colony (Figure 2(e)), but this suppression was not obvious on 1/2TSA (Figure 2(f)). Interestingly, the growth of *S. mutans* was inhibited severely when FB-T9 was inoculated first on both types of media (Figure 2(g–h)). However, no obvious competitive inhibition was observed when *S. mutans* was inoculated first (Figure 2(i–j)), indicating that although FB-T9 could inhibit S. *mutans* under either nutrient-rich or nutrient-deficient conditions, S. *mutans* had no inhibitory effect on FB-T9.

### *S. Mutans* biofilm formation mediated by FB-T9 *in vitro*

According to a report by Nobbs and colleagues [26], the formation of an early biofilm of *S. mutans* can be observed at 24 h. The formation period includes five key time points: 0 h, initial bacterial adherence; 6 h: initial bacterial colonization; 12 h: initial early biofilm formation; 24 h: maturation of early-stage biofilm and 48 h: maturation of the later-stage biofilm. In this study, we added the FB-T9 fermentation supernatant at these five time points to mediate the formation of the *S. mutans* biofilm. The *S. mutans* biofilm biomass, total number of bacteria in the biofilm and viability of biofilm were recorded under different experimental conditions.

**Figure 3.**
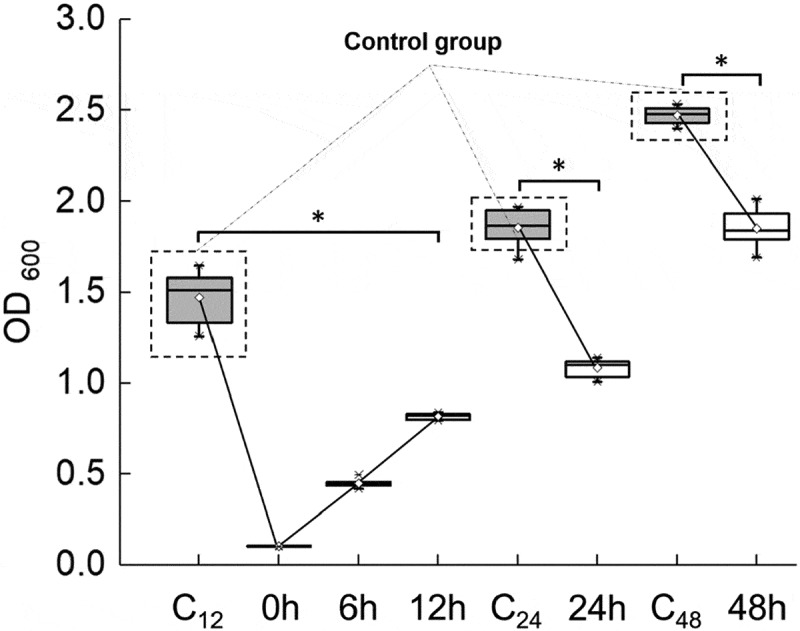
Biomass of *S. mutans* mediated by *L. plantarum* FB-T9 at different stages. C_12_, biofilm of negative control at 12 h. C_24_, biofilm of negative control at 24 h. C_48_, biofilm of negative control at 48 h. *P < 0.05 when compared with the control treatment and compared between groups

The biomass of the *S. mutans* biofilm increased gradually from 0 to 48 h (Figure 3). The addition of FB-T9 at 0, 6 and 12 h significantly influenced the formation of the biofilm (p < 0.05). The most obvious decreases were observed at 0 and 6 h. In particular, the *S. mutans* biomass at 6 h was only a third of the control value. Moreover, the addition of FB-T9 at 24 and 48 h significantly inhibited the *S. mutans* biofilm biomass (p < 0.05).

**Figure 4.**
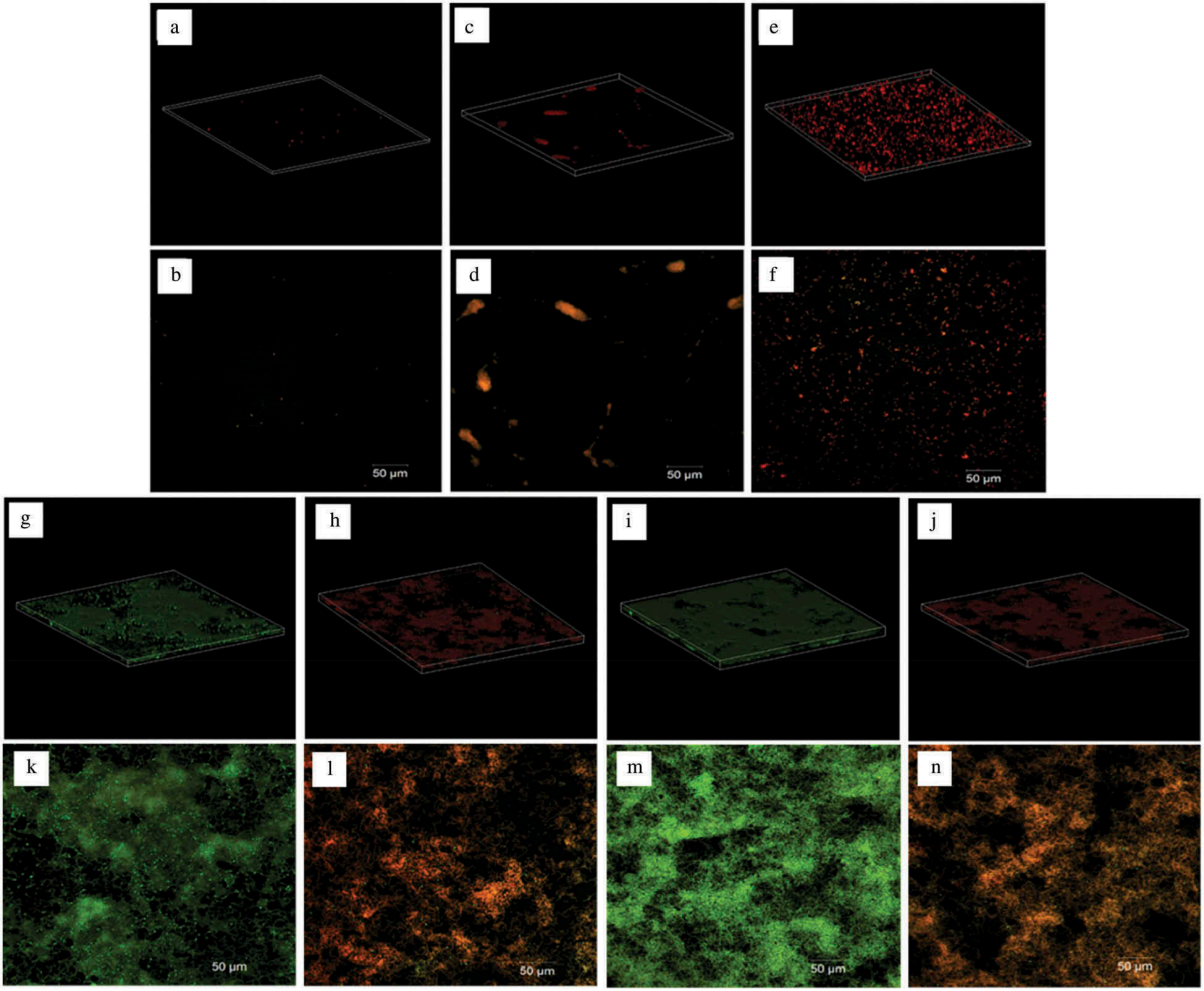
CLSM of *S. mutans* biofilm on glass coverslips. Addition of FB-T9 bacterial suspension at 0 h (A and B), 6 h (C and D), 12 h (E and F), 24 h (H and L) and 48 h (J and N) respectively. (G and K) *S. mutans* biofilm after incubation for 24 h with medium alone. (I and M) *S. mutans* biofilm after incubation for 48 h with medium alone. And A, C, E, G, H, I, J are corresponding 3D graphs

CLSM was used to visualize viable and non-viable bacteria. When FB-T9 was added to the *S. mutans* suspension at 0 h, no attached bacteria were observed after a 24-h culture (Figure 4(a–b)). Meanwhile, the areas of bacteria in the biofilms of cultures treated at 6 and 12 h increased gradually, indicating that the biofilms accumulated gradually before the intervention (Figure 4(c–f)). Additionally, the biofilms formed in the negative control group cultures (24 and 48 h) were dense and concentrated, with large areas containing viable bacteria (Figure 4(g,i,k,m)). In the cultures treated at 24 and 48 h, larger proportions of non-viable bacteria were observed, and biofilm structure was relatively loose and dispersed (Figure 4(h,j,l,n)). Visually, no obvious differences were observed between the control and mediation groups at 24 and 48 h. Hence, the total bacterial area and biofilm viability were calculated.

**Figure 5.**
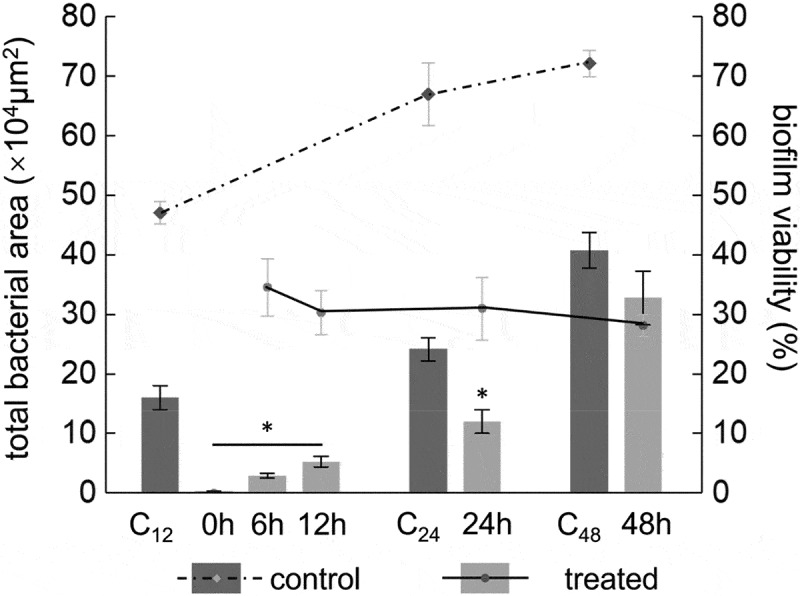
Total bacterial area of biofilm and biofilm viability after intervention by *L. plantarum* FB-T9 at different stages. The left axis corresponded to the histogram, showing the bacterial area of biofilm (×10^4^ μm^2^). Biofilm mediated by FB-T9 at 0 h, 6 h, 12 h was compared to the 12 h negative control, while biofilm mediated by FB-T9 at 24 h and 48 h were compared to the 24 h and 48 h negative control, respectively. The dashed line indicated the biofilm viability of three controls, and the solid line indicated the biofilm viability after treatment at different stages. C_12_, biofilm of negative control at 12 h. C_24_, biofilm of negative control at 24 h. C_48_, biofilm of negative control at 48 h. The assay was performed three times and data are expressed as mean ± standard error of the mean. *P < 0.05 when compared with the control treatment and compared between groups

The bacterial areas of the early-treated (0, 6 and 12 h) biofilms ranged from 0.33 to 5.34 × 10^4^ μm^2^ (Figure 5), while that of the 12-h negative control biofilm was 16.08 × 10^4^ μm^2^. The bacterial areas of the treated biofilms were significantly smaller than that of the control biofilm (p < 0.05), representing a reduction of more than 65%. Furthermore, the medium-term (24 h) bacterial area decreased by more than 50% after treatment. In the later-stage of biofilm maturation, the total bacterial areas in the 48-h mediation groups decreased only slightly when compared with the 48-h negative control, and this difference was not significant (p = 0.063). Interestingly, the viability of the biofilm decreased gradually with the time interval after the intervention, whereas the value of the negative control increased gradually. These results indicate that earlier FB-T9 mediation led to a better inhibitory effect on *S. mutans* biofilm formation (p < 0.05).

### Evaluation of a caries-induced rat model

#### Bacterial colonization of the rat oral cavity

No significant differences in weight gain and visceral coefficients were observed between the groups of rats (p > 0.05, data not shown), and all animals appeared to be in good physical condition throughout the experiment. The concentrations of *S. mutans* on the molars of the dental caries model group and the intervention group ranged from 1 to 3 × 10^5^ CFU/mL (Table 1), indicating that *S. mutans* had successfully colonized the oral cavities after 5 days of infection. When chlorhexidine was applied for another 5 days (second sampling), the *S. mutans* concentration decreased to 0.1 × 10^5^ CFU/mL and stabilized at 0.2 × 10^5^ CFU/mL at the end of the treatment (third sampling). However, population of *S. mutans* after FB-T9 treatment decreased two orders of magnitude from 3.2 × 10^5^ to 0.03 × 10^5^ CFU/mL (third sampling) which showed a greater extent than chlorhexidine (p < 0.05). Interestingly, treatment with 5D-3 also reduced the population of *S. mutans*, although it could not inhibit biofilm formation *in vitro* (data unpublished). Briefly, no significant differences in the effects of the interventions were observed between the three groups at the second and third samplings.

**Table 1.** *S. mutans* counts from rat dental samples at different periods

	Caries-free group (CFU/mL)	Caries group(×10^5^CFU/mL)	Intervention group(×10^5^CFU/mL)		
Sampling times	C3	C	T1	T2	T3
1	<30^Aa^	2.56 ± 0.7^Ba^	3.37 ± 0.8^Bb^	3.20 ± 2.2^Bb^	1.23 ± 0.36^Bb^
2	<30^Aa^	1.89 ± 0.8^Ba^	0.10 ± 0.0^Ca^	0.04 ± 0.0^Ca^	0.06 ± 0.0^Ca^
3	<30^Aa^	1.40 ± 0.7^Ba^	0.20 ± 0.0^Ca^	0.03 ± 0.0^Ca^	0.15 ± 0.0^Ca^

A, B and C indicated the difference in the number of *S. mutans* in the oral cavity of rats in each row.

a, b and c showed the difference of the number of *S. mutans* in the oral cavity of rats sampled three times in each group.

Data are presented as the mean with the standard error of the mean in parentheses following the statistical analyses of all pairs using the Tukey–Kramer multiple comparison test (*n* = 8).

**Figure 6.**
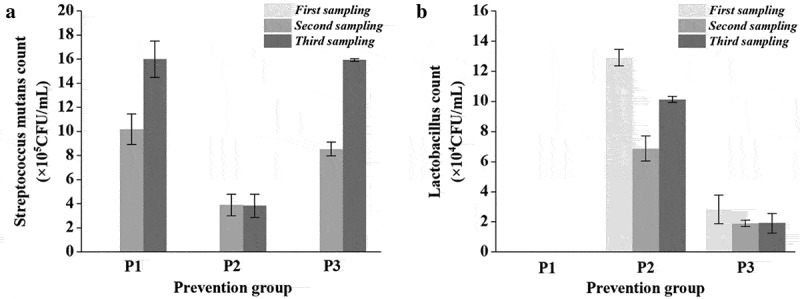
*S. mutans* & lactobacilli count from rat dental samples at different periods in prevention groups. (A) *S. mutans* counts; (B) lactobacilli counts; Data are expressed as mean ± standard error of the mean (n = 8)

To evaluate the preventive effects of lactobacilli, rats were initially inoculated with lactobacilli for 5 days and then continuously infected with *S. mutans* for 5 days. Compared with the initial *S. mutans* infection (Table 1), the *S. mutans* population in prevention groups decreased by 1–2 orders of magnitude (Figure 6(a), second sampling). In particular, the population of *S. mutans* in the FB-T9 prevention group (P2) was significantly lower than that in the other two groups (p < 0.05) at the second sampling and remained at the same level after 2 months (third sampling). In contrast, the number of *S. mutans* in the other two prevention groups increased significantly (p < 0.05) during the experiment (P1, P3).

Initially, FB-T9 colonized the oral cavity to a concentration of approximately 10^5^ CFU/mL (Figure 6(b)), which was significantly higher than that induced by 5D-3 (10^4^ CFU/mL). Meanwhile, the number of FB-T9 initially decreased in the second sampling but increased in the third sampling. It was speculated that FB-T9 may successfully colonize the oral environment via a competitive process. However, 5D-3 did not undergo a similar process, indicating that FB-T9 was generally retained better than 5D-3 in the rat oral environment.

#### Caries lesion evaluation by Keyes scoring

The cheek, tongue and adjacent surfaces and sulcal of a rat molar can be divided into several dental caries units according to the extent of caries damage, as shown in Table 2. Whereas Table 2 demonstrates how the dental caries units were determined and scored, the score results for the corresponding caries are shown in Table 3. Dentin caries (Dm) occurred in the model and treatment groups but not in the prevention groups, whereas deep dentin caries (Dx) did not occur in any experimental groups. The FB-T9 prevention group (P2, 14.7) and intervention group (T2, 23.9) received the two lowest total dental caries scores (E; 14.7 and 23.9, respectively). Both of these scores were less than half of the score in the model group, indicating that FB-T9 significantly prevented and treated dental caries *in vivo*. Overall, caries reduction was more efficient in the prevention groups than in the intervention groups. However, the effect in the 5D-3 prevention group (P3) did not surpass that in the chlorhexidine prevention group (P4), and 5D-3 (T3) had no significant treatment effect (p > 0.05). Interestingly, the Dm score in the FB-T9 group was significantly lower than that in the model group (p < 0.05), whereas no significant difference was observed between the chlorhexidine and model groups (p > 0.05). FB-T9 appeared to have a better therapeutic effect with respect to deep caries, whereas chlorhexidine had a more superficial therapeutic effect.

**Table 2.** Number of linear units assigned to each molar

	Mandible			Maxillary		
Caries site	1st	2nd	3rd*	1st	2nd	3rd*
Buccal	6	4	4	6	4	3
Lingual	6	4	4	6	4	3
Sulcal	7	5	2	5	3	2
Proximal	1	2**	1	1	2**	1

*1st, 2nd and 3rd denote the first, second and third molars, respectively;

**The second molar contains a near-middle and a far-middle neighbourhood.

**Table 3.** Effect of different groups on caries development (incidence and severity) in rats

Group	Number	Caries level		
		E	Ds	Dm
Caries group	C3	47.6 ± 0.7	28.6 ± 1.0	15.3 ± 1.3
Intervention group	T1	34.8 ± 1.4*	24.7 ± 1.3*	14.7 ± 0.9
	T2	23.9 ± 1.0*	14.4 ± 0.9*	8.6 ± 0.8*
	T3	40.7 ± 1.3	27.6 ± 1.1	13.5 ± 0.7
Prevention group	P1	28.0 ± 0.8*	10.6 ± 0.7*	–
	P2	14.7 ± 0.9*	8.0 ± 0.6*	–
	P3	30.7 ± 1.5*	14.5 ± 0.8*	–

Data are expressed as mean ± standard error of the mean (*n* = 8). E, enamel caries; Ds, dentin exposed; Dm, three-fourths of the dentin affected. * *P* < 0.05.

## Discussion/conclusion

Dental caries is associated closely with cariogenic bacterial species, such as *S. mutans*. Although it is impossible to eradicate such microorganisms completely, interest in the potential use of probiotics to prevent oral caries has been increasing in recent years. The inhibitory effects of probiotics against *S. mutans* may be due to antimicrobial substances produced by probiotics, including organic acids, hydrogen peroxide, bacteriolytic enzymes, bacteriocins and biosurfactants [27,28]. Organic acids and hydrogen peroxide can enable probiotics to tolerate certain acidic conditions and to survive in an oral environment containing a certain amount of lysozyme [3]. According to Figure 2, when FB-T9 was spotted first on a culture plate, it had a stronger inhibitory effect on *S. mutans* than when *S. mutans* was spotted first, implying that substances produced by FB-T9 may contribute to its effects against *S. mutans*.

Oral cavity is a nutrient fluctuation environment which creates scarce resources such as nutrients and space for bacteria, so they are more likely to metabolize some inhibitory substances to suppress the surrounding microorganisms to ensure their nutritional needs and survival, which involves a mechanism called quorum sensing [29,30]. Nutritional limitations and environmental fluctuations stimulate the synthesis of signal molecules in bacterial cells. When the concentrations of these signal molecules reach a threshold, appropriate target genes are transcribed and used to produce active substances, thus inhibiting competitive bacteria [31,32]. Kreth *et al*. [33] observed that the nutritional richness can interfere with the inhibitory capacity of *Streptococcus sanguinis* against *S. mutans*. In general, under nutrient-rich conditions, the magnitudes of inhibition were higher than under nutrient-limited conditions, except for a few species in specific environmental conditions. One can consider that under nutrient-limited conditions, the energy expenditure of the bacteria was employed for bacterial survival and not for the production of H_2_O_2_ [34]. In our study, we use 1/2 TSA to simulate the condition of nutrient deficiencies and observed the interaction between *S. mutans* and *Lactobacillus*. The result showed that FB-T9 not only exerted a strong inhibitory effect on *S. mutans*, the main cariogenic bacteria, but also exhibited a strong competitive advantage under nutrient-deficient conditions. Therefore, we may infer that FB-T9 is a strong competitor against *S. mutans* for temporal and spatial niches.

Dental caries is well established as a representative biofilm-dependent oral disease. Although *S. mutans* is not always the most abundant bacteria in the oral cavity, it is a key producer of matrix and coordinator of cariogenic biofilm formation [35]. Many studies have proven the effects of *Lactobacillus* on *S. mutans* biofilms at 24 h [36–38]. To date, however, the mechanism by which *Lactobacillus* interferes with the formation of *S. mutans* biofilms at different stages and the correlation between the biofilm biomass and activity at different stages remains unknown. In this study, *S. mutans* biofilm formation was mediated by FB-T9 at five key time points (0, 6, 12, 24 and 48 h), as described above. The following key indicators were used: (i) crystal violet staining to characterize the biofilm biomass, (ii) total bacterial area and proportions of viable and non-viable bacteria and (iii) biofilm structure. The results showed that earlier mediation yielded better effects against the biofilm. In addition, almost no biofilm formation was observed at 0 h mediation (Figure3-5(a)), possibly because the initial adhesion of *S. mutans* was inhibited by *Lactobacillus*. FB-T9 remained effective even against an initially maturing or already mature *S. mutans* biofilm, as demonstrated by the inactivation of all bacteria in the mediated biofilm. Lin *et al*. [15] also used fluorescence staining to determine the viability of *S. mutans* biofilm after the intervention of five *Lactobacillus* strains, which indicated the inhibitory effect of five *Lactobacillus* strains on *S. mutans* biofilm. Moreover, the structure of the mediated biofilm in our study was looser than that of the control, suggesting that *Lactobacillus* had a significant effect at 48 h (Figure 4). We speculated that although *S. mutans* had formed a mature biofilm at 48 h, *Lactobacillus* inhibited the proliferation of *S. mutans* in the dense biofilm and destroy the dense, cross-linked biofilm structure. Many biofilm structures contain channels through which environmental fluids can move. These channels often act as transport vessels to deliver nutrients, remove waste products and serve as conduits for messenger molecules [39]. Destroying the biofilm structure will affect the physiological activity of the biofilm. The analysis of CLSM suggested that the effect of biofilm inhibition is not necessarily to reduce the bacterial area of biofilm, but also to destroy the structure of biofilm and reduce its viability.

The inhibitory effects of *Lactobacillus* on cariogenic bacteria and biofilm formation observed *in vitro* do not necessarily mean that *Lactobacillus* has the same effects *in vivo*. Therefore, this study further examined the anti-caries activity of FB-T9 in a rat model of dental caries. *L. plantarum* 5D-3 that exhibited poor inhibitory activity against biofilms in previous experiments (data unpublished), was selected as control. During a 70-day experimental period, *S. mutans* counts and caries scores used to evaluate the decay process demonstrated that the number of *S. mutans* decreased from 10^5^ to 10^3^ CFU/mL after intervention (Table 1). In each intervention group, the number of *S. mutans* differed significantly in the second and third sampling related to the first sampling (p < 0.05), indicating significant reductions in the population of *S. mutans*. In particular, no *S. mutans* was detected in the second and third samplings after FB-T9 mediation. In Figure 6(a), the number of *S. mutans* after FB-T9 colonization was significantly lower than the number in the other two groups, suggesting that FB-T9 had more effectively prevented the colonization of *S. mutans* than had chlorhexidine and 5D-3. Meanwhile, the FB-T9 prevention group received the lowest caries score of all of the groups (Table 3). As shown in Figure 6(b), FB-T9 appeared to better prevent caries because of its strong ability to colonize the rat oral cavity. Previous studies showed that *L. rhamnosus* SD11 was detected in large quantities in the oral cavity even 4 weeks after the cessation of consumption and had a significant inhibitory effect on the population of *S. mutans* in the oral cavity [40,41], which is similar to our findings. The ability of a probiotic to colonize the oral cavity is key to its function, and this ability is strain-specific. For example, 5D-3 may effectively prevent biofilm formation, perhaps because it had the advantage of pre-colonization of the oral cavity (Figure 6(a,b) and Table 3). Hence, the ability of *Lactobacillus* spp. to colonize oral cavity should be considered an indicator of oral probiotics. FB-T9 exhibited excellent efficacy in both the prevention and treatment groups and demonstrated that a potential probiotic could outperform chlorhexidine in the treatment of dental caries (Table 3). Current studies have shown that probiotic products can reduce the population of *S. mutans* in the human oral cavity [42,43].

In conclusion, this study enabled us to identify *L. plantarum* FB-T9 as a highly suitable probiotic. This bacterium antagonized *S. mutans in vitro*, inhibited biofilm formation and reduced viability in the biofilm at different stages of formation. Moreover, the *in vivo* animal experiments revealed that FB-T9 could significantly reduce the population of *S. mutans* in the rat oral cavity, as well as the caries score. It appears that *L. plantarum* FB-T9 is a potential new oral probiotic, which can be further developed into a probiotic preparation with practical application value to prevent dental caries.
